# Tumor immune microenvironment characterization and response to anti-PD-1 therapy

**DOI:** 10.7497/j.issn.2095-3941.2015.0022

**Published:** 2015-06

**Authors:** Mariacarmela Santarpia, Niki Karachaliou

**Affiliations:** ^1^Medical Oncology Unit, Human Pathology Department, University of Messina, Messina 98122, Italy; ^2^Translational Research Unit, Dr Rosell Oncology Institute, Quirón Dexeus University Hospital, Barcelona 08028, Spain

In recent years, further understanding of the interaction between the immune system and tumor growth has led to the development of several immunotherapies. These immunotherapies include cancer vaccines and immune checkpoint inhibitors that have been tested in various solid tumors, including those traditionally considered non-immunogenic, such as non-small cell lung cancer (NSCLC). In physiological state, T-cell-mediated immune response against foreign antigens is regulated by stimulatory and inhibitory signals, which are critical to prevent autoimmunity and protect normal tissues after immune system activation. Cancer cells harbor different genetic and epigenetic alterations; thus, neoantigens that are potentially recognized and eliminated by the immune system are expressed. Adaptive immune responses, particularly IFN-γ-secreting T cells, exert a core function in tumor immune surveillance. However, tumors can escape this surveillance and maintain an immunosuppressive microenvironment through multiple mechanisms, including recruitment of regulatory cells (e.g., regulatory T cells, myeloid-derived suppression cells, and type 2 macrophages) and production of molecules suppressing antitumor T-cell responses (e.g., interleukin-10 and transforming growth factor-β). Tumor growth is also associated with immunomodulation of T-cell response through enhancement of co-inhibitory molecules or immune checkpoints, such as cytotoxic T-lymphocyte-associated antigen-4 (CTLA-4) or programmed cell death-1 (PD-1), on T cells^1^^,^^2^. Immunotherapies may affect various tumors by activating adaptive immune system response, including blockade of immune checkpoint pathways.

**Figure 1:**
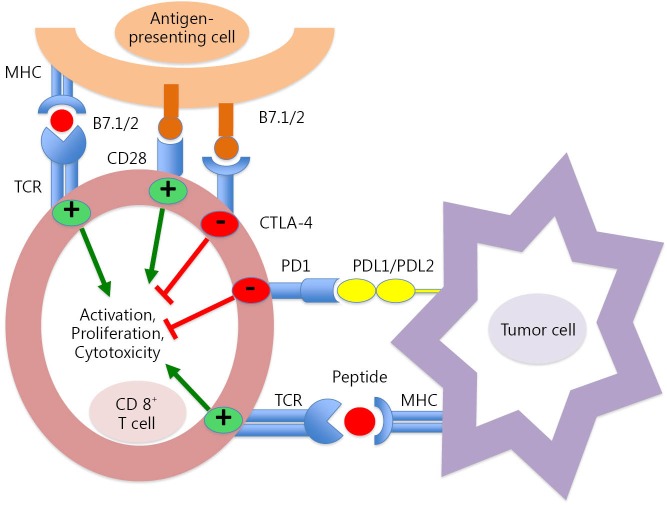
The programmed cell death 1 (PD-1)/PD-L1 pathway. MHC, major histocompatibility complex; TCR, T-cell receptor; CTLA-4, cytotoxic T-lymphocyte antigen-4.

PD-1 receptor is highly expressed by activated T cells, B cells, and natural killer cells^3^. The well-known ligands of PD-1 are PD-L1 (or B7-H1) and PD-L2 (or B7-DC). PD-L1 is expressed in macrophages and can be induced by inflammatory cytokines on tumors, immune cells, and various tissues (**Figure 1**). After ligand binding, PD-1 inhibits kinase signaling pathways involved in T-cell activation; thus, this process prevents overstimulation of immune response. PD-L1 also binds CD80 receptor, which is another negative regulator of T-lymphocyte activation. PD-1 primarily inhibits T-cell activity in the effector phase within tissues and tumors, whereas CTLA-4 regulates immune responses early in T-cell activation. Therefore, PD-L1/PD-1 axis blockade should enhance anticancer immunity. Antibodies directed against CTLA-4 and PD-1/PD-L1 pathway have been demonstrated to be effective treatment strategies, which induce durable tumor responses in patients with various malignancies^4^. Several anti-PD1 antibodies, including nivolumab (BMS-936558), have been developed and are currently in advanced phases of clinical development; nivolumab is a full human IgG4 monoclonal antibody that binds to PD-1 receptor and can block interaction with both of its ligands. Nivolumab disrupts negative signaling triggered by PD-L1/PD-L2 and restores T-cell antitumor function. In a phase I nivolumab study, patients’ cumulative response rates (at all doses from 0.1 to 10 mg/kg every 2 weeks) were 18% in NSCLC, 28% in melanoma, and 27% in renal-cell cancer. Grade 3 or 4 adverse events were observed in 14% of patients, with three drug-related deaths caused by pneumonitis^5^. In a phase II trial that enrolled patients with advanced squamous NSCLC, who had received two or more prior treatments, nivolumab was associated with 14.5% response rate after an 11-month follow-up, with 3.3-month median response onset. Responses were durable, with 77% of responders who presented ongoing responses during analysis^6^. CheckMate 017, a phase III trial, was stopped early in January 2015 following an assessment conducted by the independent Data Monitoring Committee; this committee concluded that the study reached its endpoint, demonstrating superior overall survival with nivolumab compared with docetaxel in patients with advanced squamous NSCLC pretreated with platinum-based chemotherapy. The abovementioned trials included patients regardless of their PD-L1 status. First-line treatment with nivolumab conferred a significant improvement in overall survival and progression-free survival compared with dacarbazine, with low risk of high-grade toxic effects, in a phase III randomized trial including patients with metastatic melanoma without BRAF mutation^7^. In the current study, secondary endpoints included correlation of tumor PD-L1 expression with overall survival.

Nivolumab provides significant clinical benefits in different cancer types; thus, identification of predictive response factors is crucial to select patients who will most likely benefit from treatment, while sparing resistant patients from unnecessary toxicity. PD-1/PD-L1 is predominantly involved in the final stages of immune response between T cells and tumors; therefore, most biomarker investigations have focused on tumor microenvironment. Specifically, several studies have explored PD-L1 expression in tumor cells from patients treated with PD-1/PD-L1 axis inhibitors, and significant antitumor activity has been shown in patients with PD-L1-positive tumors, although responses have also been observed in PD-L1-negative patients^5^^-^^10^.

To clearly observe the complexity of mechanisms involved in tumor response to PD-1 blockade among different tumor types, Taube *et al*.^11^ have explored the predictive value of PD-L1 expression and multiple immune biomarkers of tumor microenvironment in nivolumab-treated patients. Specifically, they analyzed the features of tumor-infiltrating immune cell subsets; they also observed PD-1, PD-L1, and PD-L2 expression in tumor cells and immune cells through immunohistochemistry (IHC) in pretreatment samples from patients with advanced treatment-refractory solid tumors treated in phase I multi-institution trial of nivolumab in the Kimmel Cancer Center at Johns Hopkins University. The said trial evaluated the interrelationship among the factors, as well as potential correlations with clinical outcomes in patients who had received at least three biweekly nivolumab doses and demonstrated evaluable treatment responses. IHC for PD-L1 and PD-1 was performed with 5H1 and M3 mAbs, respectively; samples were considered “positive” if cell surface PD-L1 and PD-L2 expression by tumor cells and tumor-infiltrating immune cells, including tumor-infiltrating lymphocytes (TILs) and histiocytes, was ≥5%. Immune infiltrate intensity (scored from 0 to 3) was correlated with the proportion of tumor cells expressing PD-L1. The proportion of TILs expressing PD-1 was also analyzed.

A total of 68 pretreatment archival or newly obtained tumor samples from 41 patients with advanced melanoma, NSCLC, renal-cell carcinoma, colorectal carcinoma, and castration-resistant prostate cancer (CRPC) were analyzed. PD-L1 expression varied among tumors and mainly occurred in tumor cells of melanoma, NSCLC, and kidney cancer specimens, in contrast to only one colorectal and no CRPC specimens. Tumor PD-L1 expression was significantly correlated with infiltrating immune cells, including lymphocytes and histiocytes (*P*=0.001), and the proportion of PD-L1-positive tumor cells correlated with infiltrate intensity (*P*=0.003). PD-L1 expression was also observed in TILs and histiocyte/macrophages among different tumor types, including some colorectal cancer specimens, in which tumoral PD-L1 expression was very low. Interestingly, the receptor PD-1 expression in TILs was significantly associated with the expression of its ligand, PD-L1, in tumor cells and immune cell infiltrates; this finding suggested the presence of a potentially immunosuppressive environment. Furthermore, PD-L2 function in immunosuppression mediation was explored in the present study. PD-L2 was less expressed than PD-L1, but its interaction with PD-1 was also blocked by nivolumab. Only 8 (21%) out of 38 specimens showed PD-L2 expression in tumor or infiltrating immune cells, which were mainly associated with PD-L1 expression.

To assess the predictive function of pathological features in patients with multiple specimens (16/41, 39%), an analysis was conducted by considering only the specimen closest to nivolumab initiation or that with maximum expression of each variable. Tumor PD-L1 expression was significantly associated with objective response (*P*=0.025), as assessed by RECIST 1.0, and clinical benefit (objective response or stable disease ≥6 months; *P*=0.005). PD-L1 expression by immune cells was significantly correlated with clinical benefit only. Considering the presence of TILs correlated with tumor cell PD-L1 expression, such marker was analyzed as an independent potential predictive factor, although this marker was not correlated with favorable clinical outcomes to nivolumab. All other microenvironment factors were analyzed, including PD-1 and PD-L2 expression; CD4:CD8 ratio, CD20^+^ B cells, and tumor necrosis of lymphoid aggregates were not correlated with response to treatment. Correlation of PD-L1 expression with clinical outcome was not related to the timing of tissue acquisition (ranging from 0 to 13 years).

Taube *et al*.^11^ provided novel interesting insights into interactions among different factors of tumor microenvironment and their function in mediating response to anti-PD-1 therapy. First, PD-L1 expression in tumor cells emerged as the strongest predictive factor associated with response to nivolumab. A robust association between tumor PD-L1 expression and response to PD-1 pathway blockade has been reported in many studies. PD-L1/PD-L2 tumor expression is important in PD-1 receptor activation among activated T cells, thereby suppressing T-cell-mediated immune response and creating a local immunosuppressive milieu. However, Taube *et al*. suggested that PD-L1 expression does not exclusively represent an immunosuppressive factor; such marker may mainly reflect an ongoing antitumor immune response characterized by an immune-activated microenvironment and the production of various cytokines and inflammatory factors (e.g., IFN-γ) that induce PD-L1 and PD-L2 expression. PD-L2 was correlated with PD-L1 expression, but pure PD-L2 showed no correlation with response to nivolumab or other PD1/PD-L1 agents^10^^,^^11^.

Importantly, PD-L1 expression patterns and characteristics of immune infiltrates can vary among tumor types, and these factors can affect response to PD-1 blockade. Majority of melanoma, kidney cancer, and NSCLC showed PD-L1 expression in tumor cells, which were also correlated with immune infiltrate intensity in melanoma and kidney cancers. By contrast, PD-L1 expression occurred in infiltrating immune cells; however, this process was absent in tumor cells among colorectal cancers. As underlined by the authors, these distinct expression patterns may reflect heterogeneity of immune response among various tumors, which can be influenced by specific genetic changes within the tumor and by other tumor stromal components. Tumor cells express various neoantigens, including those associated with somatic mutations or infection by tumor-promoting viruses, which can activate a local inflammatory response that is potentially responsible for PD-L1 up-regulation. In some cancer types, PD-L1  expression has been associated with activation of downstream signals, including PI3K/AKT, PTEN, and ALK/STAT3 pathways; therefore, this process may target immune response in some oncogene-addicted tumors^11^^-^^14^. PD-L1 expression has also been demonstrated to be either a negative or positive prognostic biomarker in different tumor types^15^^-^^18^.

The presence of TILs, which have been correlated with chemotherapy or immunotherapy activity in some advanced cancers, may be necessary to drive PD-L1 expression in tumors. However, in the present study, TILs were not independently correlated with response. This study also reported the association of PD-L1 expression by infiltrating immune cells with clinical benefits from nivolumab, although tumor PD-L1 expression was the most important factor associated with objective response. In a recent study on patients with different cancer types treated with anti-PD-L1 monoclonal antibody, MPDL3280A, the association of PD-L1 expression by infiltrating immune cells in pretreatment samples with objective response was stronger than that with tumor cell PD-L1 expression; therefore, these cells may be involved in a pre-existing T-cell activity suppression by PD-L1 before therapy. This pre-existing immunity can serve a crucial function in determining response to treatment with MPDL3280A^10^.

Different studies significantly reported that some rates of objective response to PD-1 pathway blockade have been observed in patients with PD-L1-negative tumors; thus, PD-L1 expression may not be the only predictive factor for this class of inhibitors^5^^-^^10^. The survival benefit from first-line nivolumab versus dacarbazine was observed across all pre-specified subgroups of metastatic melanoma patients, including subgroups defined by PD-L1 status^7^. Results in terms of PD-L1 expression predictivity are difficult to interpret because studies significantly vary with regard to the use of different antibodies for IHC analysis, different cutoff levels used to define positivity, and different methods of sample collection, processing, and storage. In some nivolumab studies, PD-L1 expression was evaluated using anti-PD-L1 monoclonal antibody 5H1 (threshold for positivity: 5% expression per specimen), whereas others used an automated IHC assay based on a sensitive and specific anti-PD-L1 monoclonal antibody, 28-8 (positivity: ≥5% tumor cells showing membrane staining of any intensity in a section containing at least 100 tumor cells). Sample size can also affect the results because of the focal nature of PD-L1 expression in tumors and known intratumoral genetic heterogeneity. Furthermore, given the dynamic nature of antitumor response, site (anatomical location), and time of collection (primary or metastatic cancer), biopsy can affect the expression of PD-L1 and other immunologic factors. However, in the trial conducted by Taube *et al*.^11^, no significant correlations existed between biopsy characteristics and PD-L1 expression. In some studies, result interpretation was also limited by the small number of patients analyzed. Overall, these data suggest that patients with PD-L1-negative tumors should not be considered ineligible for these highly effective immunotherapeutic approaches. The predictive function of PD-L1 expression and other immunoarchitectural features of pretreatment tumors will be specifically addressed in ongoing phase II and III trials on PD-1/PD-L1 pathway inhibitors in different solid tumors, including melanoma and NSCLC. Additional studies on tumor microenvironment factors and their relationship with PD-L1 expression are needed to better elucidate the different clinical activities of immunotherapies across tumor types. In melanoma, other immunological events seem to affect response to treatment, such as pre-existing CD8^+^ T cell infiltration at the invasive tumor margin, which is associated with the expression of PD-1 and PD-L1 immune inhibitory axis and correlated with response to pembrolizumab^19^. Furthermore, in melanoma, pretreatment tumors in responding patients demonstrated elevated IFN and IFN-inducible gene expression (e.g., CXCL9); these associations were not found in NSCLC or renal cancer^10^. Recent studies have demonstrated that genomic tumor landscape can predict response to immune checkpoint inhibitors. Some tumors, including NSCLC and melanoma, are characterized by very high mutational burdens. Neoantigens created within these tumors can trigger and construct immune responses that can be enhanced by immunologic checkpoint blockade. NSCLC specimens from patients treated with the anti-PD-1 pembrolizumab were characterized by whole-exome sequencing; a higher mutational load was also strongly associated with clinical efficacy^20^. Moreover, efficacy is correlated with a molecular smoking marker, specific DNA repair pathway mutations, and higher burden of candidate neoantigens. Similarly, in melanoma patients treated with ipilimumab, a long-term clinical benefit was associated with high mutational load^21^. However, some tumors with high load of somatic mutations failed to respond to checkpoint blockade; therefore, other tumor characteristics may affect therapeutic benefit. In the same study, researchers identified a set of neoantigens that were common to patients who presented a sustained clinical benefit; however, these neoantigens were completely absent in patients with minimal or no benefit. This specific marker in tumors can be potentially useful to select patients who will most likely benefit from CTLA-4 blockade.

Our knowledge about the mechanisms involved in the interactions between the immune system and tumors is rapidly evolving. In this context, the work of Taube *et al*. provided a crucial contribution to elucidate how these mechanisms can be exploited to predict response to anti-PD-1 therapy and facilitate further investigations in this field.
